# Antibiotic treatment for Tuberculosis induces a profound dysbiosis of the microbiome that persists long after therapy is completed

**DOI:** 10.1038/s41598-017-10346-6

**Published:** 2017-09-07

**Authors:** Matthew F. Wipperman, Daniel W. Fitzgerald, Marc Antoine Jean Juste, Ying Taur, Sivaranjani Namasivayam, Alan Sher, James M. Bean, Vanni Bucci, Michael S. Glickman

**Affiliations:** 1 Immunology Program, Memorial Sloan Kettering Cancer Center, New York, New York, USA; 2 Infectious Diseases Service, Department of Medicine, Memorial Sloan Kettering Cancer Center, New York, New York, USA; 3 Weill Cornell Medical College, New York, New York, USA; 4 Clinical and Translational Science Center, Weill Cornell Medical College, New York, New York, USA; 5 GHESKIO Centers, Port-au-Prince, Haiti; 6 Immunobiology Section, Laboratory of Parasitic Diseases, National Institute of Allergy and Infectious Diseases, National Institutes of Health, Bethesda, Maryland USA; 7 Department of Biology, Program in Biotechnology and Biomedical Engineering, University of Massachusetts Dartmouth, Dartmouth, Massachusetts USA

## Abstract

*Mycobacterium tuberculosis*, the cause of Tuberculosis (TB), infects one third of the world’s population and causes substantial mortality worldwide. In its shortest format, treatment of TB requires six months of multidrug therapy with a mixture of broad spectrum and mycobacterial specific antibiotics, and treatment of multidrug resistant TB is longer. The widespread use of this regimen makes this one of the largest exposures of humans to antimicrobials, yet the effects of TB treatment on intestinal microbiome composition and long-term stability are unknown. We compared the microbiome composition, assessed by both 16S rDNA and metagenomic DNA sequencing, of TB cases during antimycobacterial treatment and following cure by 6 months of antibiotics. TB treatment does not perturb overall diversity, but nonetheless dramatically depletes multiple immunologically significant commensal bacteria. The microbiomic perturbation of TB therapy can persist for at least 1.2 years, indicating that the effects of TB treatment are long lasting. These results demonstrate that TB treatment has dramatic effects on the intestinal microbiome and highlight unexpected durable consequences of treatment for the world’s most common infection on human ecology.

## Introduction

Each year, up to 3–4% of all deaths worldwide from any cause are attributable to infection with the bacterial pathogen *Mycobacterium tuberculosis* (*Mtb*), the causative agent of Tuberculosis (TB) disease, which amounts to almost 5,000 TB-related deaths each day^1^. This colossal disease burden necessitates a thorough understanding of both the pathogenic strategies *Mtb* uses to cause disease, as well as the host susceptibilities *Mtb* has evolved to exploit. Individuals can be *Mtb* uninfected, infected with latent *Mtb*, have active TB disease, or be cured through antibiotic therapy. Many factors can influence the probability that some individuals transition from one of these stages to another, but most defined risk factors compromise immune function^2^. For example, untreated HIV infection, which depletes CD4+ T cells, is associated with elevated risk of TB disease. Overall, immune status is also affected by age—the elderly and young infants are at a disproportionately high risk of *Mtb* infection and subsequent TB disease. Furthermore, individuals with germline mutations in pathways involved in controlling mycobacterial infection, such as IFNγ and TNFα, have an increased risk of active TB disease^3^. Despite these examples, known immune deficiencies are not sufficient to explain why the incidence of new active TB cases hovers over 10 million people each year, with a mortality rate between 1.5–2 million people^1^. Furthermore, it is unknown why some individuals in TB endemic countries (where *Mtb* exposure is common) never become *Mtb* infected, why most latently infected individuals never progress to active TB disease, and what may account for treatment failure, relapse, and re-infection.

One emerging factor that remains unstudied in the context of TB susceptibility and treatment is the intestinal microbiota. The organisms comprising the intestinal microbiota account for the largest exposure of the immune system to the environment, and in turn, the composition and metabolic activities of the intestinal bacterial community (collectively called the microbiome) directly participate in the development and function of peripheral immunity^4^. One example of this microbiome-immune axis is the regulation of peripheral immune function through the production of short chain fatty acids (SCFAs) by the intestinal microbiota. SCFAs like butyrate and propionate are produced by commensal microbiota primarily in the gut, and can have systemic effects. Butyrate inhibits histone deacetylases causing increases in *Foxp3* ^+^ T_reg_ cell proliferation and circulation in the periphery^5, 6^. Propionate binds to GPCR41, dampening allergic reactions by reducing dendritic cell-mediated Th2 responses^7^. Similarly, babies with depletion of four microbiota constituents are at risk for asthma, and transfer of these four bacteria to germ free mice ameliorates Th2-mediated airway inflammation^8^. A recent example of how these factors may influence Tuberculosis infection comes from a study suggesting that butyrate-producing bacteria in the lung microbiota are positively correlated with increased incidence of active Tuberculosis. Thus, the microbiota, and their metabolic activity, may play an active role in immunity to *Mtb* ^9^.

Although intestinal microbiome composition can be determined by many factors throughout a lifetime, it is increasingly well-understood that, in the absence of antibiotic perturbation, it remains relatively stable^10^. The human intestinal microbiota taxonomic composition is dominated by Firmicutes and Bacteroidetes, somewhat lower levels of Actinobacteria and Proteobacteria, as well as low abundance but important phyla like Verrucomicrobia, Fusobacteria, and Euryarchaeota^11^. Antibiotics can target any of these taxa, and have distinctive microbiome-altering effects both during and post treatment. Important examples include reduced diversity after fluoroquinolone treatment^12, 13^, altered microbial carbohydrate metabolism in response to β-lactams^14^, as well as altered bile-acids, dipeptides, alcohols and fatty acids in response to third-generation cephalosporins^15^. In people undergoing cancer treatment, treatment with metronidazole led to substantial derangement of the microbiota through its anti-anaerobic activity; in contrast, treatment with intravenous vancomycin had relatively little impact^16^. Although the pre-treatment ecological state of the microbiome generally recovers after stopping antibiotic treatment, there are noticeable effects that may persist for weeks, months, and even years after treatment is stopped^17^. Although these are just a few examples, the current model is that antibiotic treatment can result in the establishment of an alternative state that could have systemic, and potentially deleterious, consequences for immunity and disease susceptibility^11^.

Little is known about the effects of first-line TB antibiotics on the intestinal microbiome. In contrast to commonly used broad spectrum antimicrobials, most first-line antibiotics used to treat TB are narrow spectrum agents with *Mycobacteria-*specific targets (Supplementary Figure S1). A standard course of TB therapy for drug-sensitive *Mtb* consists of the administration of four drugs for two months, Isoniazid (H), Rifampin (R), Pyrazinamide (Z), Ethambutol (E), and then the continuation of HR for an additional four months, as recommended by the World Health Organization. Of the four standard TB antibiotics used in “short course” treatment (HRZE), only Rifampin, which inhibits bacterial RNA polymerase, is a broad-spectrum antimicrobial that is used for non-mycobacterial infections. The effects of this prolonged antibiotic regimen on the intestinal microbiota are unknown.

In this study and a companion study in mice^18^, we characterize the immediate and long-term effects of TB treatment with HRZE on microbial diversity, taxonomic composition, and biochemical capacity. We demonstrate the substantial and unique disruptive effects of HRZE therapy on intestinal microbiome composition using both 16S and metagenomic DNA sequencing and demonstrate that durable gut microbiomic dysbiosis is a consequence of TB treatment.

## Results

### Antimycobacterial treatment alters intestinal microbiota taxonomic composition during treatment without affecting overall diversity

It is currently unknown if and how the standard regimen of antimycobacterial HRZE therapy affects the taxonomic composition of the intestinal microbiota, as none of these drugs have been studied, alone or in combination, for their effects in humans. We used a cross-sectional enrollment design to determine if and how the intestinal microbiota changes in response to HRZE therapy in people from Haiti. All subjects with active TB were recruited from the Haitian community, had recently been diagnosed with microbiologically-confirmed *M. tuberculosis* infection, and have been on a combination regimen of HRZE antibiotics for an average of 3.4 months (Treatment group, Table 1). We also assembled two control cohorts, community members without TB infection (IGRA-, Mtb uninfected) and community members with latent TB (LTBI, IGRA+), see Table 1. For this LTBI cohort, we divided the subjects into two control cohorts (LTBI (treatment) and LTBI (Cured)) to ensure appropriate age and sex matching. Using either the DESeq or LeFSe analytical pipeline (^19, 20^ see Methods), we were unable to detect any microbiomic differences between *Mtb* uninfected and LTBI individuals (data not shown), and quantitative Permanova analysis further confirmed this finding (Supplementary Table 4, see Methods). Thus, we conclude that LTBI has no detectable effect on intestinal microbiome composition.

**Table 1:** Patient populations analyzed in this study by 16S rDNA sequencing. Data are divided into study groups described in the text. The number of subjects, average age, gender distribution, time on HRZE treatment or time since treatment, average number of 16S reads and subsequent OTUs, and Shannon diversity index are shown, if applicable. *Mtb-*uninfected controls are IGRA−, people with LTBI are IGRA+ , and are the appropriate comparator group for individuals with active TB disease. The LTBI group was divided based on the comparisons being made to Treatment and Cured groups, matching for age (see methods).

Group	Number of subjects	Average Age (range)	% female	Time on TB treatment	Time since TB treatment	Average number of 16S reads per patient (range)	Average number of OTUs per subject (range)	Shannon Diversity
*Mtb* uninfected	50	33 (19–59)	62	N/A	N/A	35951 (690–116638)	230 (19–473)	3.412
LTBI (treatment control)	25	26 (17–32)	52	N/A	N/A	41038 (4713–118110)	229 (47–470)	3.341
LTBI (cured control)	26	25 (17–31)	46	N/A	N/A	40678 (5788–111151)	243 (47–477)	3.74
Treatment	19	20 (13–32)	54	3.4 months (13–258 days)	N/A	38489 (4360–140543)	150 (57–118)	3.218
Cured	19	23 (17–27)	35	6 months	424 days (34–1202 days)	19283 (4712–118180)	239 (133–356)	3.74

**Figure 1:**
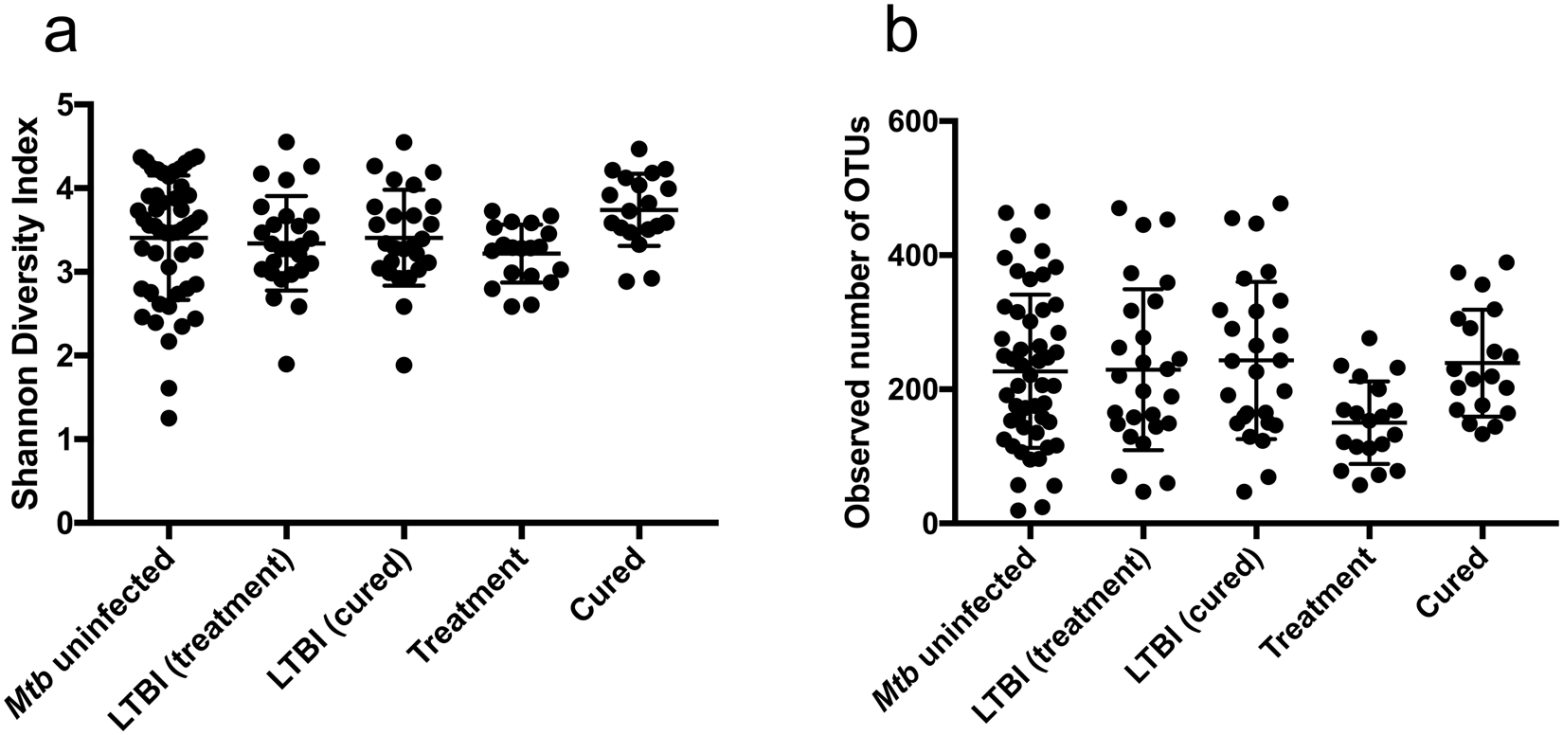
(**a**) Shannon diversity index measured for all groups used in this study, based on 16S rDNA sequencing data. The LTBI (treatment) group indicates subjects who are the age-matched controls for the treatment group, and the LTBI (cured) group indicates the age-matched controls for the cured group. (**b**) Raw number of observed OTUs clustered at 97% similarity for the indicated groups.

In comparing individuals on HRZE treatment with LTBI controls, we found that overall microbiomic diversity of subjects treated for active TB with HRZE did not differ from *Mtb* uninfected or LTBI controls, as measured by the Shannon diversity index, despite being on therapy for an average of 3.4 months (Fig. 1, Table 1). This lack of effect on diversity is in stark contrast to the dramatic and rapid loss of diversity seen with broad spectrum antimicrobials^21^, and is consistent with the narrower spectrum of antimycobacterial agents used for treatment of TB. Despite little effect on diversity, there was a highly significant loss of specific bacterial taxa with antimycobacterial treatment (i.e., the number of unique OTUs). The number of observed OTUs was significantly lower in the treatment group compared to the *Mtb* uninfected (two-samples t-test; p = 0.0074) or LTBI-treatment (two-samples t-test; p = 0.0122) control groups (Fig. 1b).

**Figure 2:**
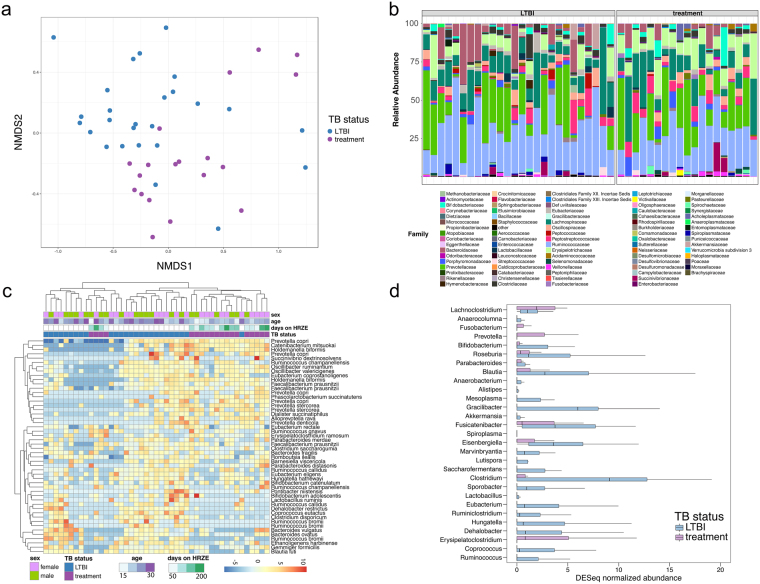
HRZE treatment perturbs the taxonomic structure of the microbiome. (**a**) NMDS ordination of HRZE treated subjects (treatment, purple) or LTBI controls (blue) based on 16S rDNA sequencing (**b**) Family taxonomic distribution of the intestinal microbiota from subjects with LTBI and subjects with TB on treatment. (**c**) Heatmap of the top 50 most abundant taxa generated with DESeq2 showing unsupervised clustering of TB cases on treatment vs. LTBI controls. Age and sex are also shown but were not accounted for the in DESeq model. Genus and species names are based on OTU identification (Supplementary Table 2) and therefore names may be redundant, but represent different 16S-based OTUs. (**d**) Taxonomic abundance profiling comparing treatment vs LTBI participants using LeFSe to determine differentially abundant Genera. Box and whisker plots of differentially abundant genera are shown based on the DESeq normalized data. Plots show the first and third quartiles of the abundance data, the line represents the median, and the whiskers show 1.5 times the value of the interquartile range.

**Figure 3:**
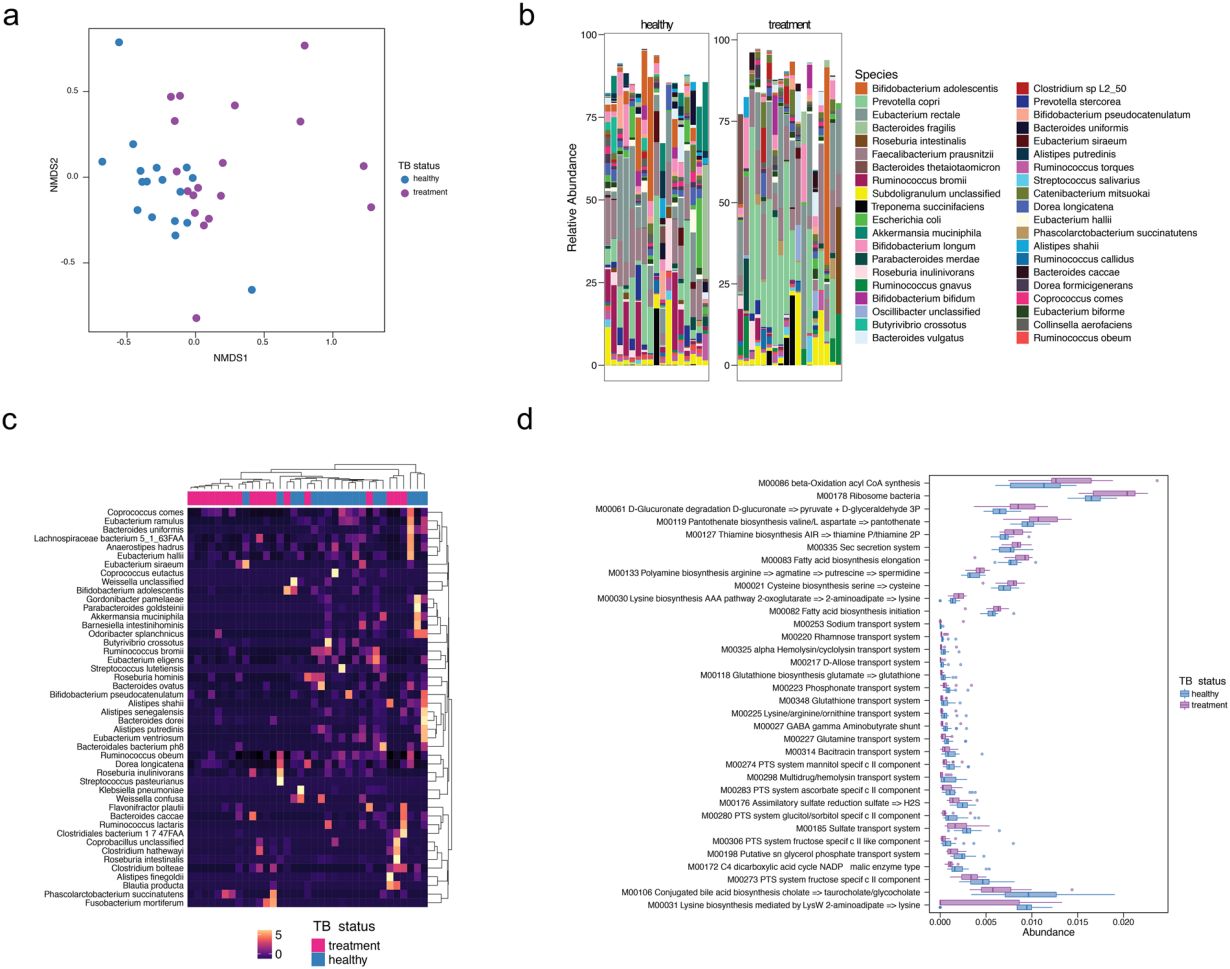
Taxonomic and biochemical microbiomic perturbation induced by HRZE. (**a**) NMDS ordination plot on metagenomic taxonomy data demonstrating microbiomic differences between healthy individuals and subjects on HRZE treatment. For this comparison, the healthy group consists of LTBI and *Mtb* uninfected subjects. **(b)** Comparative abundance plots between healthy Haitian individuals and cases on HRZE treatment showing the most abundant species. (**c**) Unsupervised hierarchical clustering of significantly altered taxa from species-level metagenomic data. (**d**) Abundance of significantly different KEGG modules between healthy volunteers and cases on treatment.

Despite the lack of an effect on overall diversity, closer examination of specific microbiomic changes associated with treatment revealed substantial changes in the treated group compared to either the *Mtb* uninfected control group or LTBI controls. Principal coordinate analysis on differential taxonomic diversity calculated either using 16S (Fig. 2a, treatment vs LTBI) or metagenomic DNA sequencing (Fig. 3a, treatment vs healthy) clearly demarcates people who are on treatment from those who are not, indicating substantial antimycobacterial-induced perturbations. A quantitative test of which variables account for variance between two groups (Permanova) identified TB treatment (p = 0.023, Supplementary Table 4, see Methods), but not gender or age (Supplementary Table 4) as the main driver. Unsupervised hierarchical clustering of the 40 most abundant OTUs highlighted similar distinctions between treated and control subjects (Fig. 2c), and revealed that treated subjects clustered into two major groups, with the predominant cluster clearly demarcated from LTBI controls (Fig. 2c).

To determine which taxa were significantly affected by HRZE therapy, we used both the LEfSe and DESeq. 2 pipelines on 16S rDNA sequencing data (see Methods) and observed dramatic changes at the Genus level. Subjects taking HRZE have, on average, an enrichment of *Erysipelatoclostridium* (8.8% in the Treatment group vs. 3.4% in LTBI controls), *Fusobacterium* (0.56% in the Treatment group vs. 0.08% in LTBI controls) and *Prevotella* (7.11% in the Treatment group vs. 3.76% in LTBI controls). HRZE treatment resulted in a 10-fold reduction in *Blautia*, a more than a 200-fold reduction in *Lactobacillus* and *Coprococcus*, and a 675-fold decrease in *Ruminococcus* compared to the LTBI group (Fig. 2d). In the Phylum Actinobacteria, there was a nearly 20-fold depletion of *Bifidobacterium* (Fig. 2d).

Although 16S rDNA based taxonomic analysis provides useful information about the relative abundances of microbiome constituents, and clearly HRZE treatment induces a profound alteration in the compositional structure of the gut microbiome, this technique cannot directly interrogate the coding capacity of the microbiota, as the gene content of taxonomically identical OTUs can differ substantially. To ask whether HRZE treatment altered metabolic coding capacity of the microbiome, we performed metagenomic sequencing and analyzed the coding capacity of HRZE treated subjects vs a control group consisting of LTBI and Mtb uninfected subjects (designated healthy in Fig. 3). Taxonomic analysis by LEfSe revealed similar depletion of *Ruminococcus*, *Eubacterium*, and other species as observed with 16S profiling, and differentially abundant species by LeFSe distinguished treated from healthy controls (Fig. 3c). Using the HUMANn2 software pipeline^22^ we observed both enrichment and depletion of biochemical pathways within the microbiome of treated subjects, with the most dramatic changes including overabundance of fatty acid oxidation and vitamin biosynthesis, and depletion of conjugated bile acid biosynthesis with treatment (Fig. 3d, Supplementary Figures S4–6). These results further confirm that HRZE treatment broadly perturbs the taxonomic and functional structure of the microbiome.

### TB treatment is associated with a lasting intestinal microbiome dysbiosis

**Figure 4:**
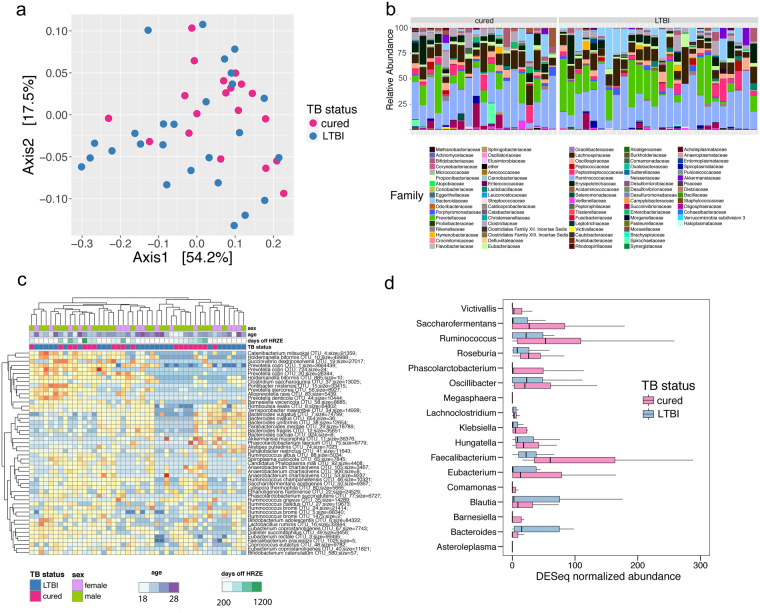
TB treatment induces a lasting alteration in microbiome structure. (**a**) DPCoA ordination plot of cured cases compared to LTBI controls based on 16S rDNA sequencing. (**b**) Family level taxonomic distribution of the intestinal microbiota from subjects with LTBI or who are cured. (**c**) Heatmap of the 40 most abundant taxa generated with DESeq2 showing unsupervised clustering of cured vs. LTBI subjects. Age and sex are also shown but were not accounted for the in DESeq model. The number of days that each patient has been off treatment is also shown. Genus and species names are based on OTU identification (Supplementary Table 3) and therefore names may be redundant, but represent different 16S-based OTUs. (**d**) Taxonomic abundance profiling comparing cured vs LTBI subjects. Taxa are significant from LeFSe (p < 0.05 and LDA cutoff >3.0).

Compelling evidence suggests that alterations in intestinal microbiome composition from antibiotics can produce novel microbiome ecological states with preliminarily characterized, but poorly defined, health outcomes^23^. The data presented above clearly indicates that HRZE treatment induces a detectable microbiomic perturbation, which could be long lasting given the prolonged duration of antibiotic exposure. To determine whether the dysbiosis induced by antimycobacterials persists after discontinuation of therapy, we recruited a group of subjects cured of TB through 6-month HRZE therapy and compared their microbiome composition to age matched LTBI subjects as controls (LTBI-Cured cohort, Table 1). The average time since completion of treatment in the cured group was 1.2 years (mean 417 days, range 34–1202 days, Table 1). We found that taxonomic alpha diversity in the cured subjects remained at levels comparable with those in the LTBI control groups (Fig. 1b). Indeed, using a Mann-Whitney unpaired t-test, there is a modest but significant increase in Shannon diversity for the cured cases (p = 0.0487, Fig. 1b). However, the intestinal microbiomes of cured TB cases were clearly distinguishable from LTBI controls when examined by double principal coordinate analysis (Fig. 4a), indicating that HRZE therapy has long-lasting effects on microbiome composition (Fig. 4b).

To quantitatively assess the double principal coordinate analysis result, a Permanova test was used to confirm that the differences between the two populations were driven by TB status (p = 0.007), rather than gender (p = 0.407) (Supplementary Table 4). Unsupervised hierarchical clustering of the 40 most abundant OTUs revealed that, in general, cured individuals clustered away from LTBI controls (Fig. 4c). We therefore asked whether the heterogeneity in the cured individuals was correlated with the duration of time since treatment and found that both distantly and recently cured people clustered well together, indicating that the persistent dysbiosis observed is not simply a marker for time since cure (Fig. 4c).

**Figure 5:**
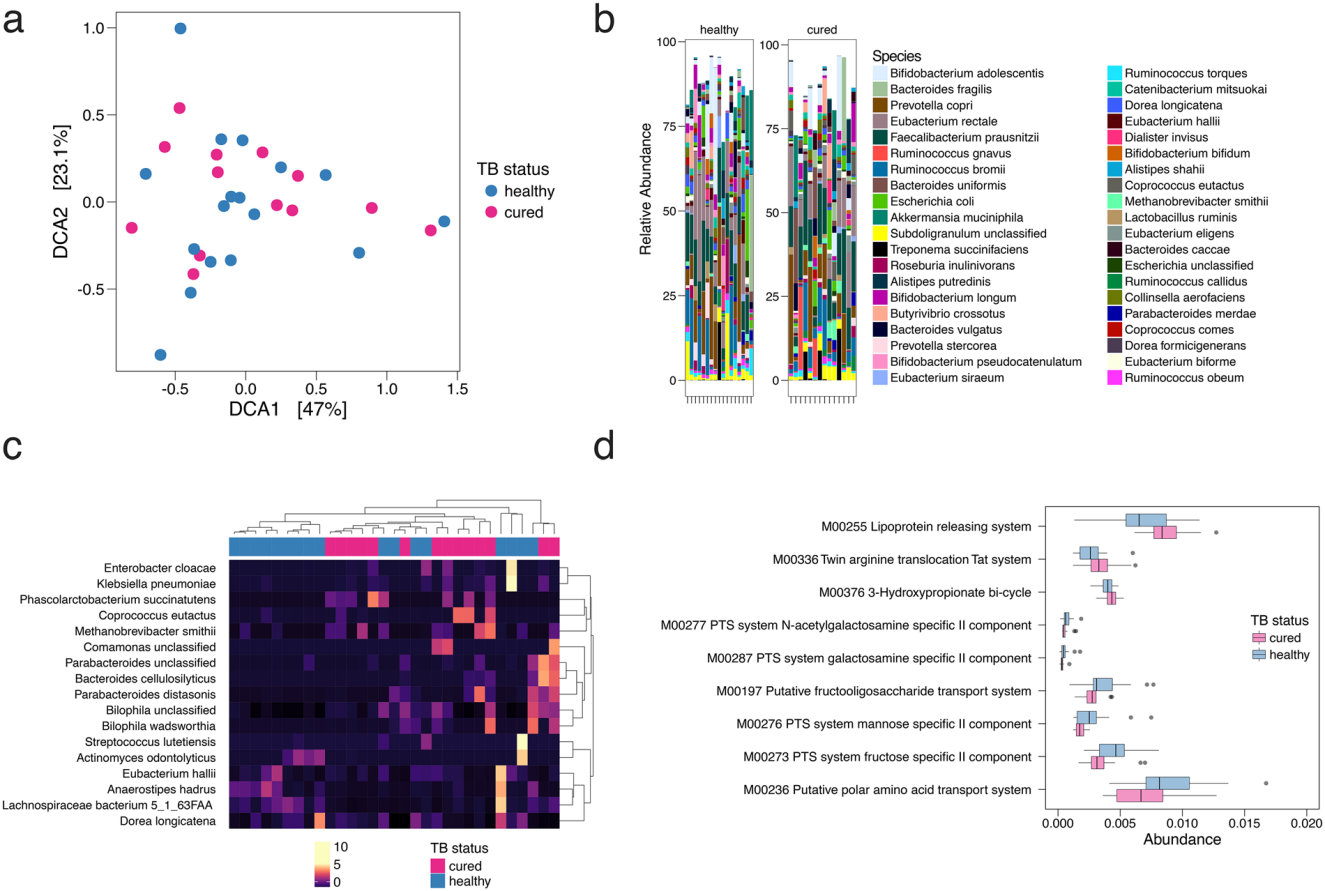
TB treatment induces a lasting alteration in microbiome structure and function. (**a**) DCA ordination plot on metagenomic taxonomy data in healthy (combined *Mtb*-uninfected and LTBI community controls) and cured individuals. (**b**) Comparative abundance plots between healthy Haitian individuals and cured subjects showing the top 40 most abundant species between the two groups. (**c**) Unsupervised hierarchical clustering of significantly altered taxa. (**d)** Abundance of significantly different KEGG modules between healthy and cured subjects.

We used LeFSe and DESeq to analyze differences between cured individuals and LTBI age matched controls. Cured people were depleted in the Bacteroidetes genera *Bacteroides* and displayed prominent overabundance of *Faecalibacterium*, *Eubacterium*, and *Ruminococcus* (Fig. 4d). Although detrended coordinate analysis (DCA) performed with species level abundances on metagenomic data failed to cluster healthy and treated cases (Fig. 5a), and community structure was grossly similar (Fig. 5b), using LeFSe, we found that *Enterobacter cloacae*, *Phascolarctobacterium succinatutens*, *Methanobrevibacter smithii*, *Bilophila*, and *Parabacteroides* are biomarkers of cured individuals (Fig. 5c). Pathway abundance analysis revealed that cured cases demonstrated altered coding capacity compared to controls. (Fig. 5d and Supplementary Figures S7–9). As with the previous comparison of healthy individuals to cases on HRZE treatment, the perturbed pathways represent diverse microbial functions including sugar biosynthesis, protein secretion, and central metabolism. We conclude that TB treatment results in long term taxonomic, metagenomic, and biochemical consequences via perturbation of the intestinal microbiome.

## Discussion

We present the first characterization of the short and long term effects of standard HRZE TB antibiotic treatment on the intestinal microbiome. Antibiotics are recognized to perturb the composition of the intestinal microbiome, and their use has been associated with potentially deleterious consequences^11^. This perturbation is best documented for broad spectrum antibacterial agents which are active against wide swaths of bacterial microbiome constituents. As such, broad spectrum antimicrobials like the fluoroquinolone ciprofloxacin^17^ may cause rapid loss of overall diversity and disruption of the microbiome’s ability to resist pathogenic colonization, which can predispose to disease such as *Enterococcus* domination and *C. difficile* infection^24^. However, treatment of TB employs antimicrobial agents with narrower spectrums of activity. Although Rifampin is used for non-mycobacterial infections, Isoniazid, Pyrazinamide, and Ethambutol are only prescribed for TB and are activated by and/or target mycobacterial proteins not widely distributed throughout the bacterial Kingdom. Since the turn of the millennium, close to 50 million people have been treated with this or similar regimens, comprising almost 10 billion doses each drug, making TB treatment one of the most widely administered antibiotic combinations in the world. Understanding the effects of this antibiotic regimen on microbiome ecology thus has important implications for those treated and cured of TB worldwide.

Our data indicates that the narrow spectrum of the TB treatment regimen is reflected in the preserved overall diversity in HRZE treated cases. However, this relatively gross measure of perturbation fails to capture the profound effects of HRZE on specific components of the microbiome. Most dramatic is the depletion of multiple species of *Ruminococcus*, *Eubacterium*, *Lactobacillus*, and *Bacteroides* along with a simultaneous increase of *Erysipeloclostridium* and *Prevotella*. The consequences of this HRZE-induced taxonomic perturbation are unknown at present, but several of these bacteria have been associated with immune-inducing phenotypes relevant to TB immunity. *Bacterioides* (depleted in treated and cured subjects) polysaccharide can modulate host inflammatory responses in mice^25^. *Ruminococcus* and *Coprococcus* are two of the most dramatically depleted phyla in HRZE treated patients, and these organisms modulate peripheral cytokine production, including IL-1, and IFNγ^26^. Similarly, *Bifidobacterium*, which we find depleted in HRZE treated cases, can induce a Th17 immune response in mice^27^. Taken together, these findings suggest that the HRZE-induced perturbation of the microbiome may have significant effects on peripheral immune responses and overall systemic immune tone. These potential effects on immunity, coupled with the well-documented variability in treatment response to TB, may suggest that variability in microbiotic perturbation and peripheral immunity could affect the efficacy of TB treatment. The data presented here will now allow for testing of this hypothesis using prospectively collected cohorts of TB cases beginning treatment, with the aim to correlate their microbiomic disruption with microbiologic and immunologic markers of treatment success.

Our findings are also corroborated by a recent study^18^ that examined the effect of TB treatment in mice. During HRZ treatment in mice, a decrease in species richness is observed, similar to the significant decrease in the number of OTUs during HRZE treatment in humans. In mice, RIF is the major driver of taxonomic alteration in the intestinal microbiome, but interestingly, combination therapy gives rise to alterations not found for monotherapy of any single antibiotic. Additionally, in both mice and humans, there is a significant decrease in the number of Clostridia during treatment, including the genera *Blautia*, *Clostridium*, and *Roseburia*.

The other prominent finding from our study is the long-lasting duration of the microbiomic disruption induced by HRZE. Our cured group had completed treatment on average 1.2 years earlier, yet their microbiomes were still detectably different from age matched control subjects using unsupervised data analysis. This finding suggests that the duration of 6 months of HRZE therapy has long lasting effects on the community structure of the microbiome. Furthermore, based on data from Namasivayam *et al*.^18^, in both mice and humans there is a persistent microbiomic dysbiosis after the completion of HRZ(E) treatment. Although alpha diversity recovers, taxonomic profiling in mice and humans, and functional pathway profiling in humans, suggests that 6-month administration of HRZ(E) treatment causes persistent changes.

As is the case for the perturbation by HRZE during therapy, the consequences of these changes in cured cases will require further study. It is possible that cured individuals could be more susceptible to systemic infection due to effects of microbiotic alteration and disruption on peripheral immunity. Multiple epidemiological studies have indicated that people cured of TB are at higher risk of a second case of TB due to reinfection^28, 29^. Although multiple environmental and genetic factors likely contribute to this risk, including HIV infection, the findings in this study raise the possibility that the persistent microbiomic disruption that accompanies curative TB treatment could contribute to post-treatment susceptibility to reinfection, perhaps not just with *Mtb*, but also with other diseases associated with an altered immune state.

In summary, we have shown that TB treatment with HRZE in humans perturbs the intestinal microbiome in distinct and long lasting ways. Specific genera of bacteria are depleted during treatment and functional profiling demonstrates altered functional pathway composition. These changes, in terms of both taxonomic and metagenomic function, are protracted for more than one year after the completion of therapy for TB disease. This study should therefore stimulate additional investigations into the role of microbiomic disruption in response to TB therapy and post TB health.

## Methods

### Study Approval

All volunteers provided written informed consent to participate in this study. All protocols and consent forms have been approved by the GHESKIO and Weill Cornell Medicine institutional review boards. All methods and procedures were performed in accordance with the relevant institutional guidelines and regulations.

### Patient Recruitment and Protection of Human Subjects

Subjects were enrolled through the Tri-Intuitional Tuberculosis Research Unit (TBRU) in conjunction with the GHESKIO Centers, in Port-au-Prince, Haiti, where all participants provided written, informed consent. All TBRU protocols and consent forms for samples collected at GHESKIO were approved by Institutional Review Boards at the GHESKIO and Weill Cornell Medicine (see Study Approval). A dedicated clinical field team at the GHESKIO Centers in Port-au-Prince, Haiti recruited research volunteers as part of the NIH U19-funded Tuberculosis Research Unit (AI111143). Patient *Mtb*-infection status is determined using quantiferon IFNγ release assay (IGRA) status, and active TB disease is determined using standard clinical assessments. All cases with active pulmonary TB receive periodic follow-up appointments while on treatment, and anyone with known contact to an active TB patient receives a six-month follow-up and is re-screened for IGRA status. All patient samples were de-identified on site using a barcode system before they were shipped to NYC for analysis. Human DNA was decontaminated from metagenomic shotgun sequencing data before analysis and publication, consistent with the removal of all biometric identifiers according the Health Insurance Portability and Accountability Act^30^. All clinical metadata was collected on site and managed through the REDCap data management system^31^.

### Clinical characteristics of study groups from the TBRU study

We recruited four groups of individuals using a cross-sectional research study design. To characterize the intestinal microbiomes of individuals from the Haitian population, we recruited two groups of control individuals, 50 with no *Mtb* infection (IGRA-), and 25 latently infected by *Mtb* (LTBI), as defined by a positive Interferon Gamma Release Assay (IGRA) test. To determine the effect of HRZE antimycobacterial treatment on the intestinal microbiome, we recruited 19 volunteers currently on treatment with HRZE for drug sensitive Tuberculosis. 3 of these treated individuals were on TB therapy for longer than the standard 6 months, due to clinician discretion (see Table 1). In addition, to determine the duration of the microbiome perturbation of HRZE treatment, we recruited 19 previously treated cases who were cured of active TB. The clinical characteristics of the groups are given in Table 1. To appropriately control for age, we divided our LTBI group into two distinct control subgroups, designated LTBI (treatment control) and LTBI (cured control), since microbiome composition can vary significantly with age^32^. Given the age range of the treatment and cured patients, we used controls under the age of 33 years old for the treatment control group, and controls under the age of 30 for the cured control group. All subjects are HIV negative. However, other clinical variables, such as diabetes history, were not available.

### DNA extraction from stool

Stool specimens were collected and stored for less than 24 hours at 4 °C, aliquoted (~2 mL each), frozen at −80 °C, and shipped to NYC. ≈500 mg of stool from frozen samples was suspended in 500 μl of extraction buffer (200 mM Tris-HCl, pH = 8.0; 200 mM NaCl; 20 mM EDTA), 210 μl of 20% SDS, 500 μl of phenol/chloroform/isoamyl alcohol (25:24:1), and 500 μl of 0.1-mm-diameter zirconia/silica beads (BioSpec Products). Samples were lysed via mechanical disruption with a bead beater (BioSpec Products) for two minutes, followed by two extractions with phenol/chloroform/isoamyl alcohol (25:24:1). DNA was precipitated with ethanol and sodium acetate at −80 °C for 1 hour, re-suspended in 200 μl of nuclease-free water, and further purified with the QIAamp DNA Mini Kit (Qiagen) according to the manufacturer’s protocols, including Protein removal by Proteinase K treatment. DNA was eluted in 200 μl of nuclease-free water and sorted at −20 °C.

### 16S rDNA sequencing

Primers used to amplify rDNA were: 563 F (59-nnnnnnnn-NNNNNNNNNNNN-AYTGGGYDTAAAGN G-39) and 926 R (59-nnnnnnnn-NNNNNNNNNNNN-CCGTCAATTYHTTTR AGT-39). Each reaction contained 50 ng of purified DNA, 0.2 mM dNTPs, 1.5 μM MgCl_2_, 1.25 U Platinum TaqDNA polymerase, 2.5 μl of 10 × PCR buffer and 0.2 μM of each primer. A unique 12-base Golay barcode (Ns) preceded the primers for sample identification after pooling amplicons. One to eight additional nucleotides were added before the barcode to offset the sequencing of the primers. Cycling conditions were the following: 94 °C for 3 min, followed by 27 cycles of 94 °C for 50 s, 51 °C for 30 s and 72 °C for 1 min, where the final elongation step was performed at 72 °C for 5 min. Replicate PCRs were combined and were subsequently purified using the Qiaquick PCR Purification Kit (Qiagen) and Qiagen MinElute PCR Purification Kit. PCR products were quantified and pooled at equimolar amounts before Illumina barcodes and adaptors were ligated on using the Illumina TruSeq Sample Preparation procedure. The completed library was sequenced on an Illumina Miseq platform per the Illumina recommended protocol.

### 16S Bioinformatics Analysis

For 16S MiSeq sequencing, paired-end reads were joined, demultiplexed, filtered for quality using maximum expected error (Emax = 1), and dereplicated. Sequences were grouped into operational taxonomic units (OTUs) of 97% distance-based similarity using UPARSE^33^. Potentially chimeric sequences were removed using both de novo and reference-based methods (where the Gold database was used for the latter)^34^. Taxonomic assignments were made using BLASTN^35^ against the NCBI refseq_rna database with custom scripts^36^. Our approach allows for the identification of the top 30 taxa associated with a particular OTU, thus the taxonomic nomenclature that we use for 16S is versatile. This OTU calling data is available in Supplementary Tables 2 and 3: Supplementary Table 2 has OTU BLASTN results for the LTBI (treatment control) and Treatment cohorts, and Supplementary Table 3 has OTU BLASTN results for LTBI (cured control) and Cured cohorts. A biological observation matrix (biom)^37^ file, a taxonomy file, reference sequence file, and tree file were constructed using QIIME commands. These files were imported into R^38^ and merged with a metadata file into a single Phyloseq object^39^. Phyloseq was used for all downstream analysis of 16S taxonomic data, and plots were made with the ggplot2 package^40^.

### Shotgun Metagenomic Sequencing

Between 150 and 200 ng of DNA isolated from stool (vide supra) was sheared acoustically. Hiseq sequencing libraries were prepared using the KAPA Hyper Prep Kit (Roche). PCR amplification of the libraries was carried out for 6 cycles. Samples were run on a Hiseq 4000 in a 125 bp/125 bp paired end run, using the TruSeq SBS Kit v3 (Illumina). There were an average number of read pairs per sample of around 11 million.

### Shotgun Bioinformatics Analysis

For the analysis of shotgun metagenomic reads, sequences were first trimmed and removed of host contamination using Trimmomatic^41^ and Bowtie2^42^. Host-decontaminated reads were then profiled for microbial species abundances using Metaphlan2^43^, and for abundance of Uniref gene and KEGG orthologs, and functional pathways (Metacyc pathways, KEGG pathways, and KEGG modules) using the software pipeline HUMAnN2^22^ and in-house written scripts (available upon request). Normalized taxonomic, gene, and pathway abundances were then used for downstream statistical analysis in R. All intestinal microbiome samples were sequenced using 16S rDNA sequencing, however, only a subset of controls were sequenced using metagenomics. Due to sample size limitations, for the metagenomic DNA sequencing comparisons, we combined both *Mtb* uninfected and LTBI individuals into a healthy control group which was used as the comparator for metagenomic analyses.

### Statistical Analysis

The ability to detect differentially abundant OTUs between groups of people is critical for comparison between groups, and various methods exist and have been validated for this sort of analysis. For 16S rDNA sequencing, we employed the tools available within the Phyloseq package to manipulate the data and metadata for downstream analysis. Raw counts with taxonomy and metadata were piped into the DESeq2 package for differential abundance analysis using the negative binomial distribution assumption with zero inflation^19^. This method assumes that for many OTUs, the variance in abundance (i.e., read count) between samples or groups exceeds the mean read count (often zero). When this is true, the DESeq method can be used to transform the data so that between sample or between group differences may be compared more accurately. Homoscedastic abundance data was used to generate heatmaps in Fig. 3c and d, by applying a variance stabilizing transformation from fitted dispersion-means to transform the count data. We additionally employed the microbiome-friendly linear discriminant analysis, effect size (LEfSe) tool^20^ to detect statistically significant differences between clinical groups. This technique first employs the non-parametric Kruskal-Wallis (KW) sum-rank test between different groups of people (i.e., healthy [comprised of *Mtb* uninfected and LTBI], on HRZE treatment, or cured), followed by linear discriminant analysis to estimate the size of the effect (i.e., the degree of significant differential abundance between a particular OTU, taxa, gene, or pathway between groups). We attempted to employ both the DESeq2 and LEfSe methods, and try to emphasize where there is overlap. All figures in the paper that are related to 16S sequence analysis are plotted using the normalized and transformed abundances from the DESeq2 package. For the statistical analysis of the results from shotgun metagenomics reads, data were imported into R and converted to Phyloseq objects with custom scripts. Custom code implementing non-parametric tests (Wilcoxon-signed rank) with FDR correction (Benjamini & Hochberg method) as well as LEfSe^20^ were used to test for differential abundances for taxa, and functional pathways. For the LTBI-Treatment and LTBI-Cured comparisons p-value threshold was kept at 0.05 for both the initial Kruskall-Wallis test and the subsequent sex-matched subclasses Wilcoxon-signed rank tests. We additionally employed the Permanova and Betadisper tests using the adonis function in the Vegan package in R. Adonis partitions a distance matrix of OTU count data and runs an analysis of variance between groups of samples. Betadisper further supports this conclusion by determining if the variance between the two groups is similarly distributed. All box-and-whisker plots were generated with the ggplot2^40^ function geom_boxplot, which shows the first and third quartiles of the dataset and the median of the data in the box, the whiskers show 1.5 times the value of the interquartile range of the box hinge, and outliers are shown as dots. All other plots were made using Prism 7.

### Data Availability

All sequencing data and computer code, as well as metadata supporting the findings of this study are available from the corresponding authors upon request.

## Electronic supplementary material

Supplementary Information

SI Table 1

SI Table 2

SI Table 3

SI Table 4
