# Supplementary material for: Antibiotic treatment for Tuberculosis induces a profound dysbiosis of the microbiome that persists long after therapy is completed
Source: Sci Rep. 2017 Sep 7;7:10767. doi: 10.1038/s41598-017-10346-6 (PMC5589918; doi:10.1038/s41598-017-10346-6)
Supplement: Supplementary file 1 — Supplementary Information [file 41598_2017_10346_MOESM1_ESM.pdf]

Supplementary Text

Antibiotic treatment for Tuberculosis induces a profound dysbiosis of the microbiome that persists long after therapy is completed

Matthew F. Wipperman[1,4], Daniel W. Fitzgerald[3,5], Marc Antoine Jean Juste[5], Ying Taur[2], Sivaranjani Namasivayam[6], Alan Sher[6], James M. Bean[1,3], Vanni Bucci[7, *], Michael S. Glickman[1,2,3,*]

[1] Immunology Program, Memorial Sloan Kettering Cancer Center, New York, New York, USA

[2] Infectious Diseases Service, Department of Medicine, Memorial Sloan Kettering Cancer Center, New York, New York, USA

[3] Weill Cornell Medical College, New York, New York, USA

[4] Clinical and Translational Science Center, Weill Cornell Medical College, New York, New York, USA

[5] GHESKIO Centers, Port-au-Prince, Haiti

[6] Immunobiology Section, Laboratory of Parasitic Diseases, National Institute of Allergy and Infectious Diseases, National Institutes of Health, Bethesda, Maryland ,USA

[7] Department of Biology, Program in Biotechnology and Biomedical Engineering, University of Massachusetts Dartmouth, Dartmouth, Massachusetts, USA

*correspondence to:

Michael Glickman, 1275 York Ave New York, New York, 10065; glickmam@mskcc.org

Vanni Bucci, 285 Old Westport Road N. Dartmouth, Massachusetts, 02747; vbucci@umassd.edu

PncA Coding Capacity Analysis

To investigate mechanistically how HRZE therapy may be altering the microbiome, we reasoned that coding capacity for the gene that is known to activate pyrazinamide, PncA, may indicate the degree of perturbation one's microbiome would experience upon HRZE exposure. To search for PncA orthologs, we used the Ortholuge Database (http://www.pathogenomics.sfu.ca/orthologedb/), an online resource that is able to provide a list of orthologous genes for a given input sequence for both Bacteria and Archaea [1]. The advantage of using a database like this is that it utilizes reciprocal BLAST, which ensures that all of the genes in the output are true orthologs of the input gene. Reciprocal BLAST first uses standard BLASTp to obtain protein homologs, and then takes the top hit of the BLASTp output and re-performs BLAST against the reference genome. Only if the top hit of this second step is the input sequence does the program consider the two sequences orthologous—a standard generally accepted by biologists [2, 3]. The Ortholuge Database output is a list of protein-coding gene sequences of all true orthologs of PncA. We then obtained the nucleotide sequences using the NIH command line E-Utilities and E-Direct programs (http://www.ncbi.nlm.nih.gov/books/NBK179288/), designed for high-throughput queries [4]. Using a custom BASH script to automatically take the ortholog output from the reciprocal BLAST as input into the *esearch* and *efetch* commands in EDirect, we obtained a fasta file of all protein coding true orthologs of PncA.

Next, we used shortBRED to find a unique set of PncA markers to which metagenomic reads could be mapped. This software relies on k-mer based identification of protein families from the input PncA sequence. We used shortbred_identify.py to cluster the PncA orthologs from all Bacteria and Archaea into families and identify k-mers, then used shortbred_quantiy.py to map metagenomic reads from the Haitian metagenomic samples to the PncA k-mer database.

The output was quantified using Prism 7 and is shown in Supplementary Figure S2. We were unable to detect any statistically significant difference in the coding capacity of the microbiomes of relevant cohorts in our study. This indicates that the microbiome coding capacity for PncA for each cohort is comparable. We thus conclude that perturbations induced by pyrazinamide, while possibly caused by the production of pyrazinoic acid, may also have other as yet undescribed mechanisms.

How the Actinobacteria Phylum is affected pre, during, and post treatment

Given the difficulty in predicting the effects of HRZE treatment specifically on the Actinobacteria Phylum, to which *Mtb* is a member, we plotted the relative abundance of the microbiota using 16S microbiome rDNA sequencing in our patient cohorts with LTBI, patients on HRZE treatment, and cured patients from this study. The results are presented in Supplementary Figure S3.

Supplementary References

1. Whiteside MD, Winsor GL, Laird MR, Brinkman FS. OrtholugeDB: a bacterial and archaeal orthology resource for improved comparative genomic analysis. *Nucleic Acids Res* **41**, D366-376 (2013).

2. Altschul SF*, et al.* Gapped BLAST and PSI-BLAST: a new generation of protein database search programs. *Nucleic Acids Res* **25**, 3389-3402 (1997).

3. Camacho C*, et al.* BLAST+: architecture and applications. *BMC Bioinformatics* **10**, 421 (2009).

4. Loman NJ, Pallen MJ. EntrezAJAX: direct web browser access to the Entrez Programming Utilities. *Source Code Biol Med* **5**, 6 (2010).

5. Kaminski J, Gibson MK, Franzosa EA, Segata N, Dantas G, Huttenhower C. High-Specificity Targeted Functional Profiling in Microbial Communities with ShortBRED. *PLoS Comput Biol* **11**, e1004557 (2015).

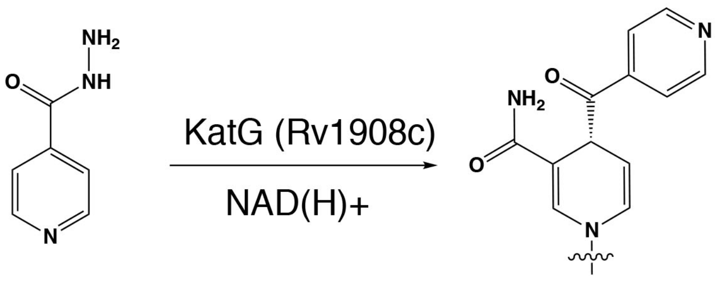

Isoniazid (H) INH-NAD

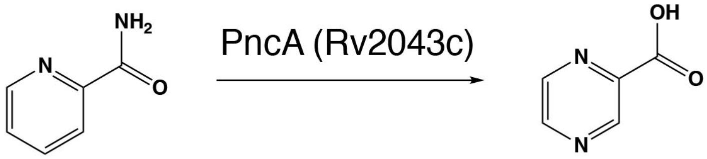

pyrazinamide (Z) pyrazinoic acid

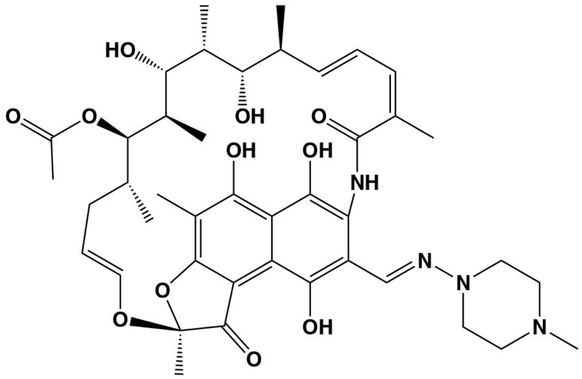

Rifampin (R)

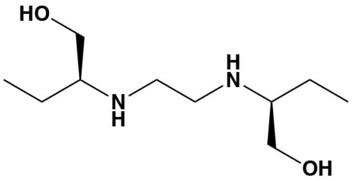

Ethambutol (E)

Supplementary Figure S1

Mechanism of action and chemical structures of HRZE drugs and their described active products. Isoniazid (H) is a prodrug, which requires the mycobacterial protein KatG (Rv1908c) for ligation with NAD+, the product of which is the active drug that inhibits enoyl-acyl carrier protein reductase InhA, inhibiting fatty acid biosynthesis. Pyrazinamide (Z) is activated by the mycobacterial enzyme PncA to pyrazinoic acid. Ethambutol (E) targets arabinogalactan biosynthesis, and Rifampicin (R) inhibits *Mycobacterial* DNA-dependent RNA polymerase through binding to the β-subunit of the enzyme.

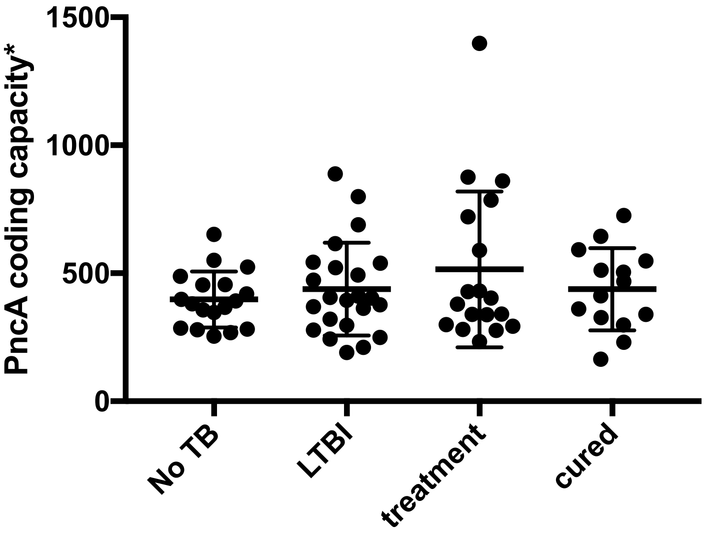

*PncA coding capacity = reads per kilobase per million mapped reads (RPKM)

Supplementary Figure S2: PncA coding capacity for each person calculated from metagenomic data and using the ShortBRED program [5]. There is no statistically significant difference between any cohort's ability to code for PncA.

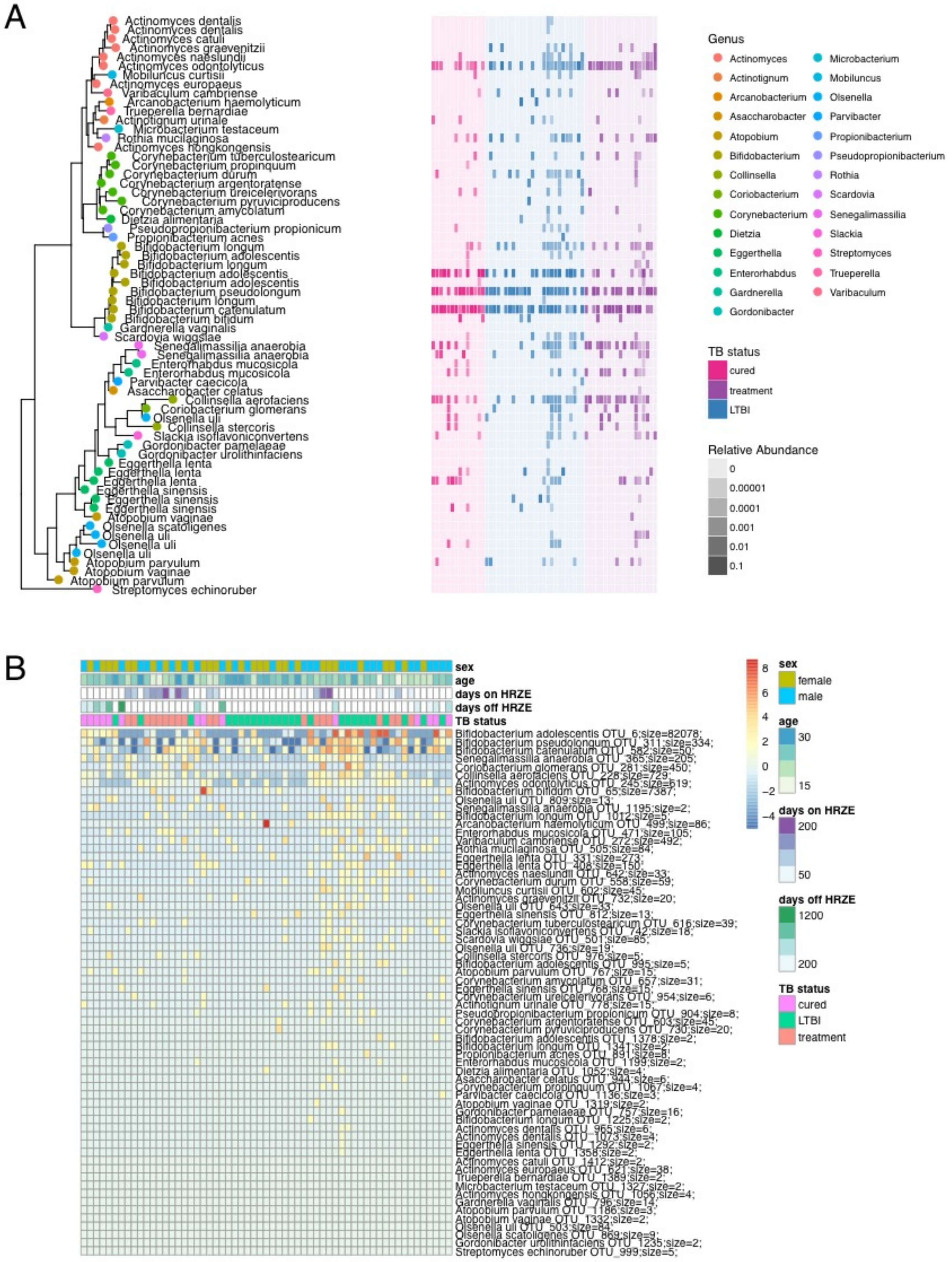

Supplementary Figure S3: A. Scaled relative abundnace of all OTUs from the phylum Actinobacteria from LTBI, treatment, and cured individuals shown on a phylogenetic tree layout. **B.** DESeq variance stabilized transformed relative abundances of Actinobacterial OTUs sampled from the intestinal microbiomes of LTBI, treatment, and cured individuals.

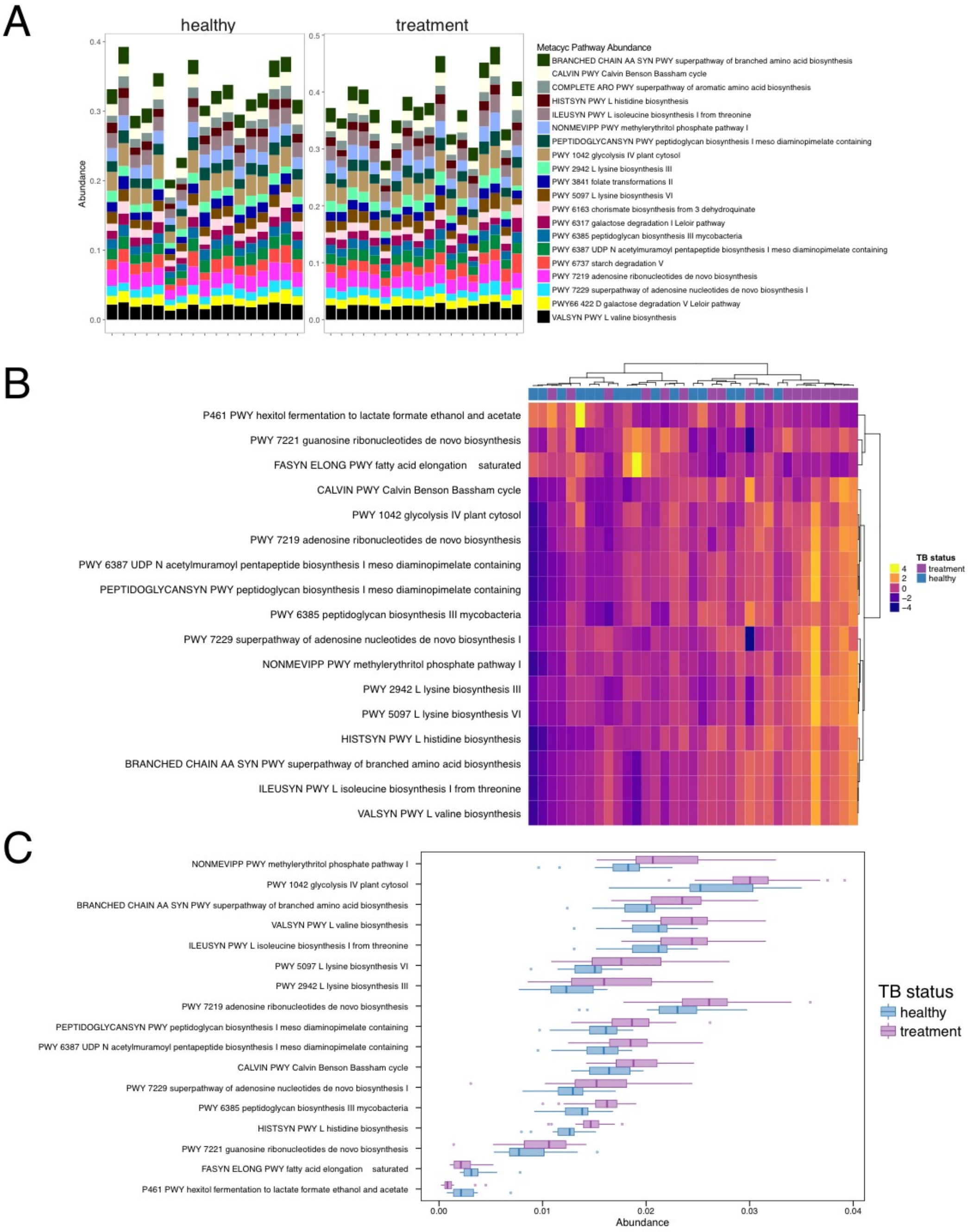

Supplementary Figure S4: Metacyc pathway abundance calculated against the UniRef50 gene database comparing healthy (uninfected and LTBI) to treated people. **A**. Top 20 most abundant pathways in the healthy and treated groups. **B**. Unsuprivised hiericherical clustering of significantly altered Metacyc pathways. **C**. Relative abundnace of Metacyc pathways, significant by LeFSe (p< 0.05), between healthy and treatment groups using Metacyc pathway coverage.

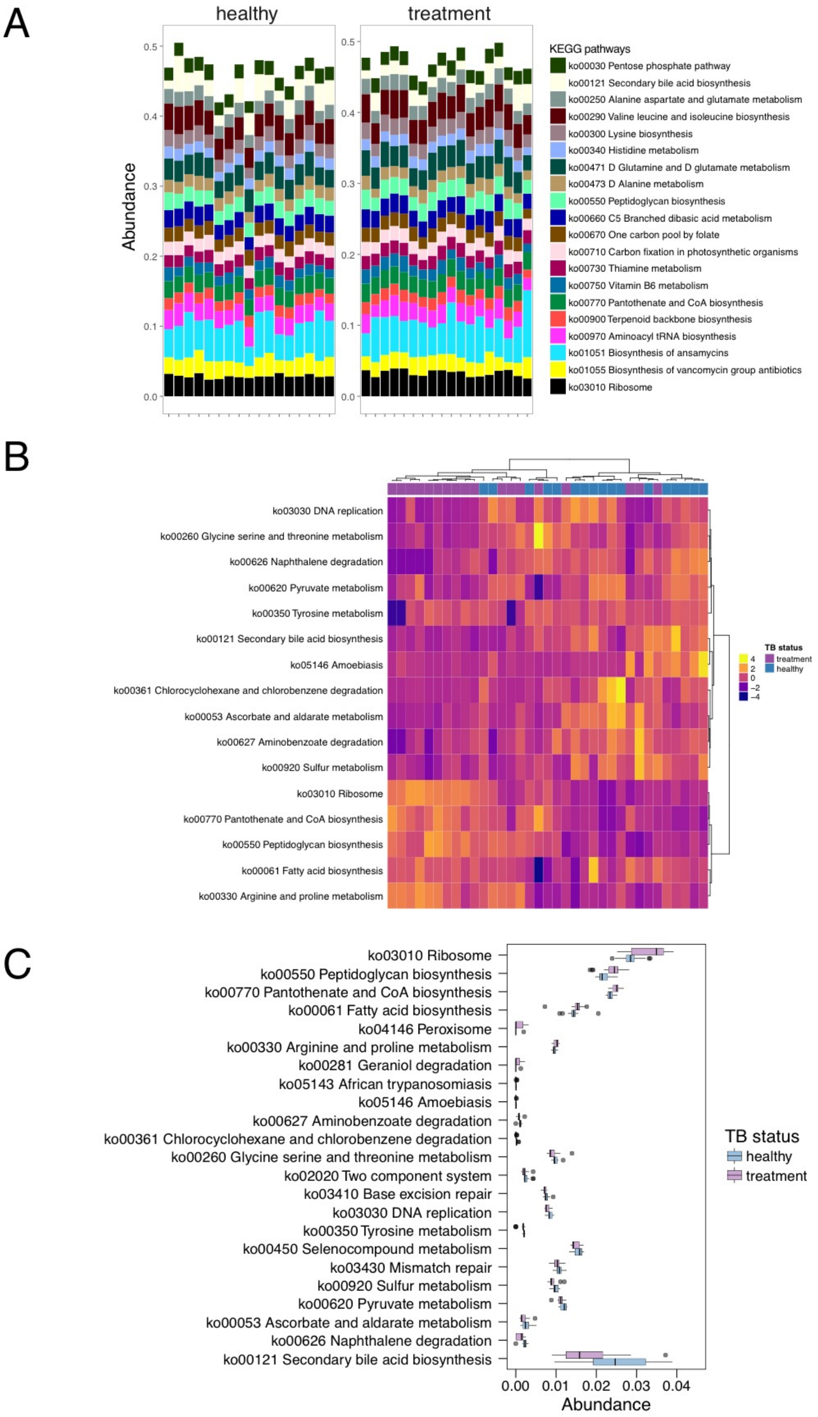

Supplementary Figure S5: KEGG pathways comparing healthy (uninfected and LTBI) to treated people. **A**. Top 20 most abundant pathways in the healthy and treated groups. **B**. Unsuprivised hiericherical clustering of significantly altered KEGG pathways. **C**. Relative abundnace of KEGG pathways, significant by LeFSe (p< 0.05), between healthy and treatment groups using KEGG pathways.

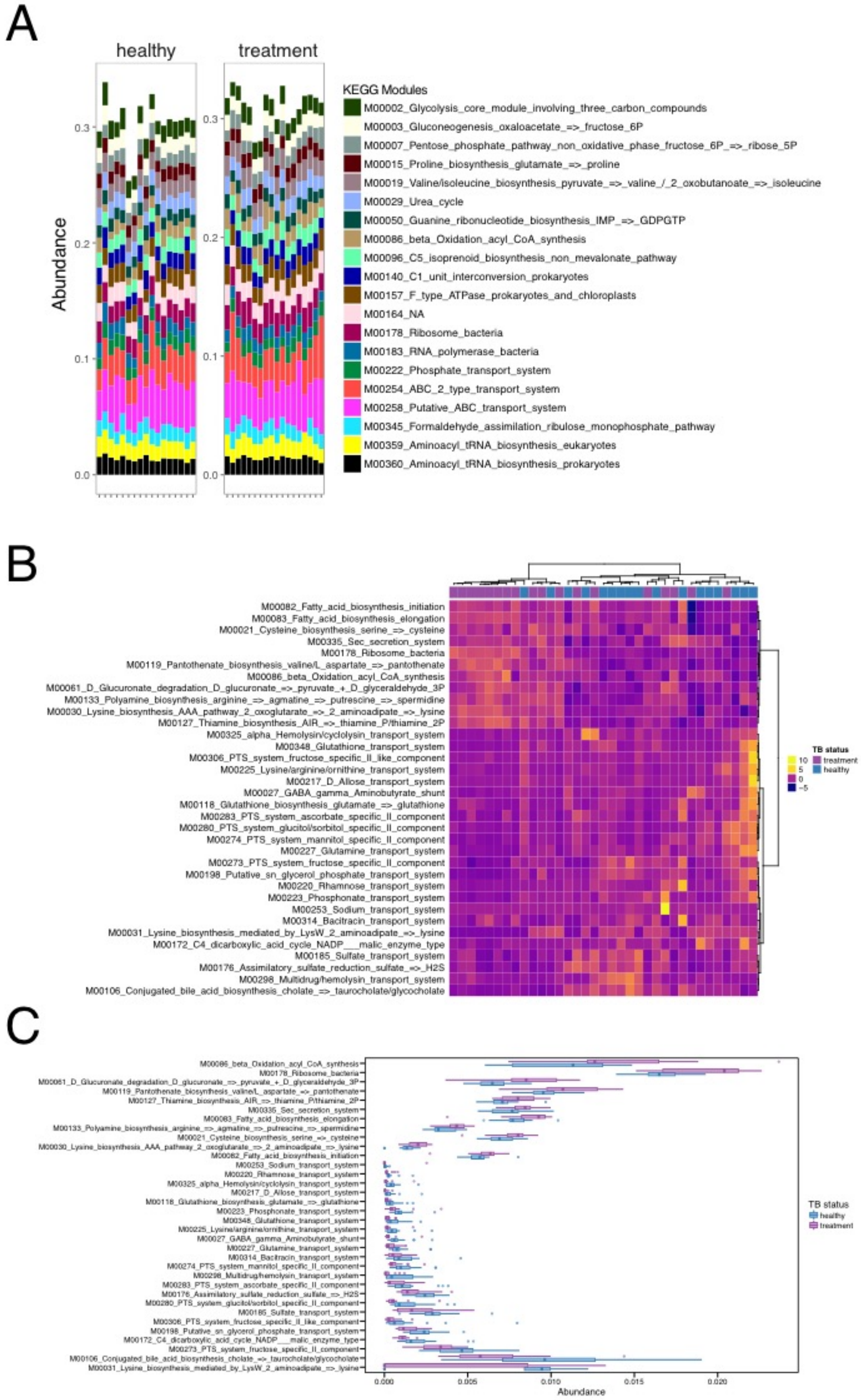

Supplementary Figure S6: KEGG modules comparing healthy (uninfected and LTBI) to treated people. **A**. Top 20 most abundant pathways in the healthy and treated groups. **B**. Unsuprivised hiericherical clustering of significantly altered KEGG modules in patients on HRZE treatmnet. **C**. Relative abundnace of KEGG modules between healthy and treatment groups.

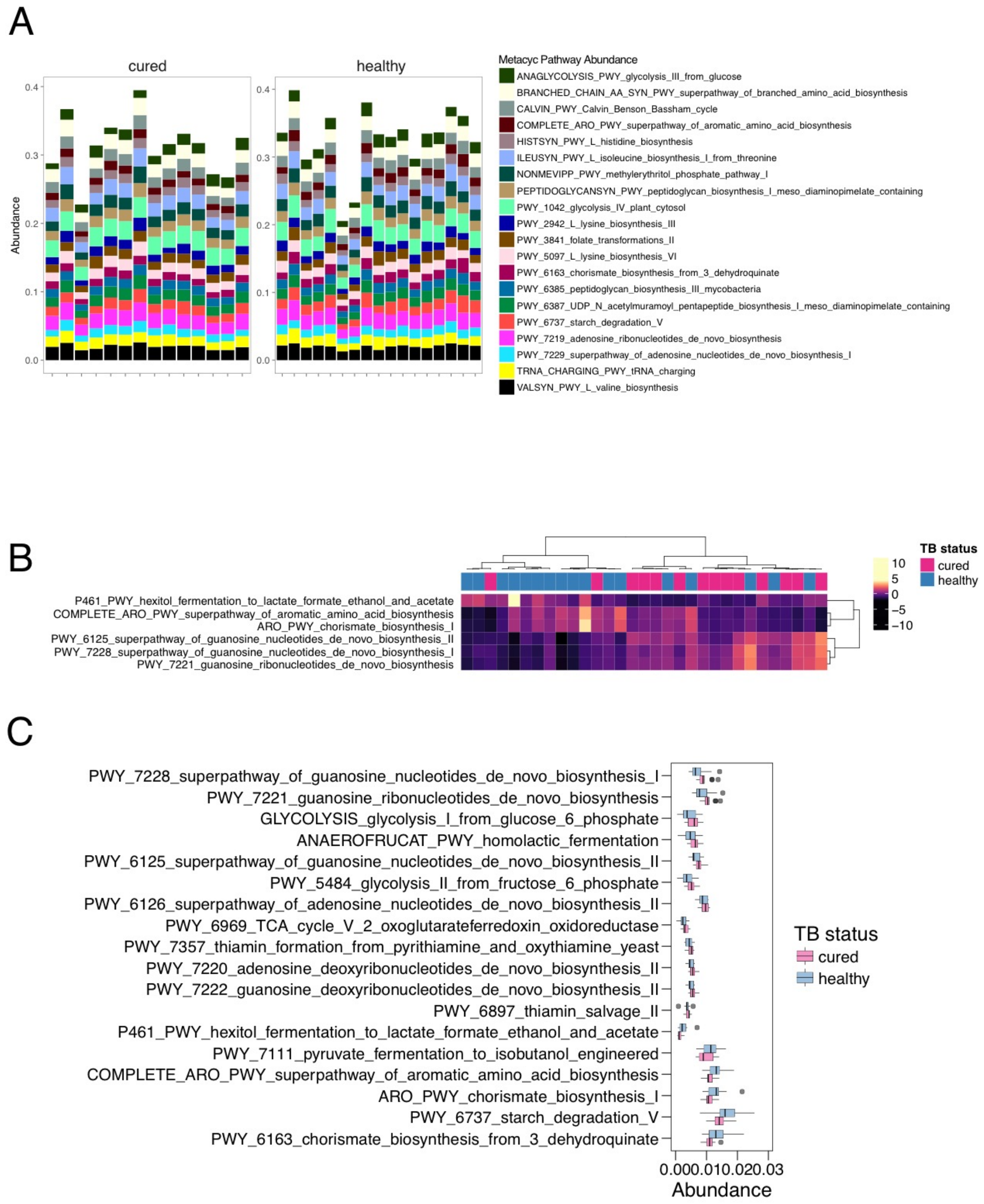

Supplementary Figure S7: Metacyc pathway abundance calculated against the UniRef50 gene database comparing healthy to cured people. **A**. Top 20 most abundant pathways in the healthy and cured groups. **B**. Unsuprivised hiericherical clustering of significantly altered Metacyc pathways. **C**. Relative abundnace of Metacyc pathways, significant by LeFSe (p< 0.05), between healthy and cured groups.

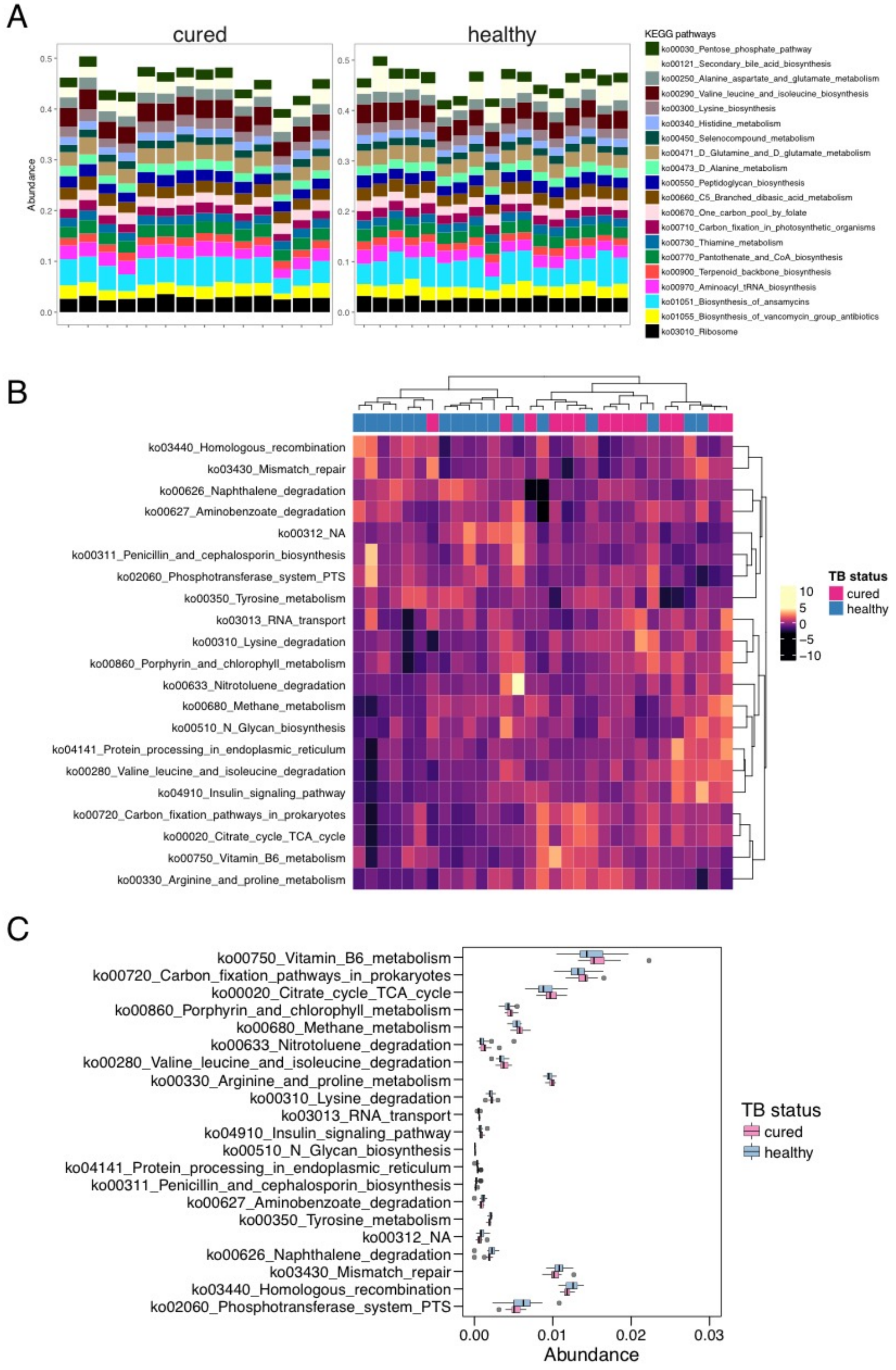

Supplementary Figure S8: A. KEGG pathways comparing healthy to cured people. **A**. Top 20 most abundant KEGG pathways in the healthy and cured groups. **B**. Unsuprivised hiericherical clustering of significantly altered KEGG pathways. **C**. Relative abundnace of KEGG pathways, significant by LeFSe (p< 0.1), between healthy and cured groups.

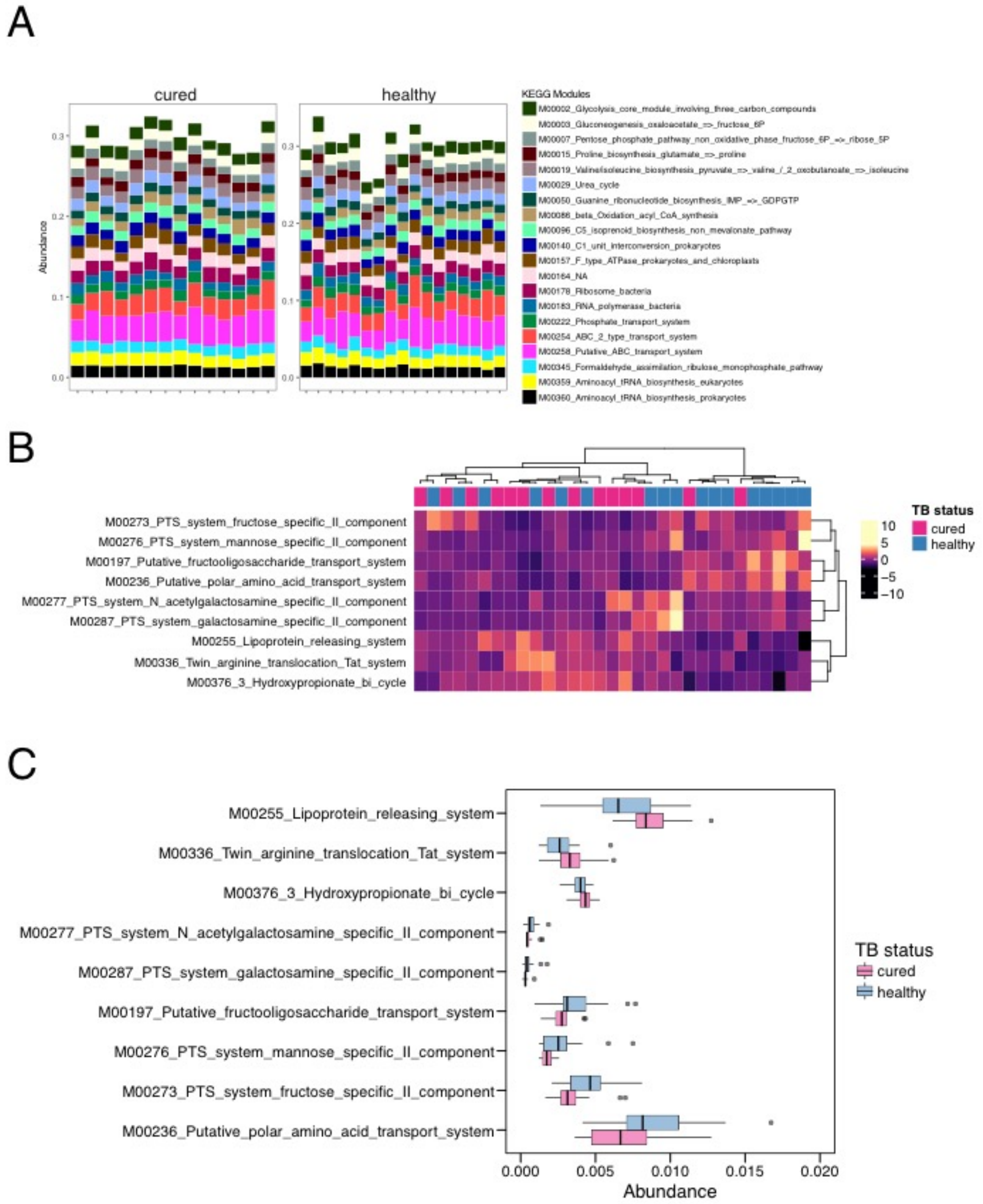

Supplementary Figure S9: A. KEGG modules comparing healthy to cured people. **A**. Top 20 most abundant KEGG modules in the healthy and cured groups. **B**. Unsuprivised hiericherical clustering of significantly altered KEGG modules. **C**. Relative abundnace of KEGG pathways, significant by LeFSe (p< 0.1), between healthy and cured groups.
